# Survival outcomes of spleen-strengthening and dampness-eliminating traditional Chinese medicine granules combined with lenvatinib for unresectable hepatocellular carcinoma: a large-sample retrospective study based on electronic medical records

**DOI:** 10.3389/fmed.2026.1946173

**Published:** 2026-10-02

**Authors:** Canchao Sun, Juan Xu, Tiye Sun

**Affiliations:** Tumor Center, Beijing Royal Integrative Medicine Hospital, Beijing, China

**Keywords:** dampness-eliminating, hepatocellular carcinoma, lenvatinib, overall survival, real-world study, spleen-strengthening, traditional Chinese medicine

## Abstract

**Background:**

Lenvatinib has been proven as a first-line systemic therapy for unresectable hepatocellular carcinoma (HCC). Traditional Chinese Medicine (TCM), particularly formulations targeting spleen strengthening and dampness elimination, has the potential for synergistic effects with targeted therapies. Researchers conducted a study to investigate the feasibility of combining spleen-strengthening and dampness-eliminating TCM granules with lenvatinib to improve survival rates in patients with unresectable HCC.

**Methods:**

This is a single-center retrospective cohort study that examined electronic medical records from one of the Beijing Royal Integrative Medicine Hospital between January 2023 and December 2025. Patients with unresectable HCC who received lenvatinib treatment as first-line therapy were divided into a combination group (TCM granules combined with lenvatinib, *n* = 186) and a control group (lenvatinib alone, *n* = 192). Balancing of baseline characteristics was done by use of propensity score matching (PSM). Overall survival (OS) was the primary endpoint; secondary endpoints included the incidence of adverse events, treatment duration, and the improvement of TCM syndromes.

**Results:**

Following 1:1 PSM, 156 patients were enrolled in each group. The combination group had a significantly longer median OS compared with the control group (18.7 months vs. 14.2 months; HR = 0.68, 95% CI: 0.52–0.89, *P* = 0.005). The incidence of Grade 3–4 adverse events was significantly lower in the combination group than in the control group (28.2% vs. 41.0%, *P* = 0.018). Treatment duration was significantly prolonged in the combination group (9.8 vs. 7.2 months, *P* = 0.012), and the combination group also showed better improvements in TCM syndrome scores.

**Conclusions:**

Adjunctive therapy with spleen-strengthening and dampness-eliminating TCM granules was associated with better survival outcomes and fewer adverse events in patients with unresectable HCC receiving lenvatinib. These findings provide a rationale for prospective trials evaluating the integration of TCM into modern oncological treatment paradigms for HCC.

## 1 Introduction

Hepatocellular carcinoma (HCC) is the sixth most commonly diagnosed cancer and the third leading cause of cancer-related death worldwide (1). The disease burden is concentrated in East Asia, where chronic hepatitis B virus infection remains a major aetiological factor. Despite improvements in surveillance and treatment, a substantial percentage of patients still present with advanced or unresectable disease, for whom curative options such as surgical resection or liver transplantation are not feasible (2).

Systemic therapy for unresectable HCC has changed substantially over the past decade, with treatment selection guided by staging systems such as the Barcelona Clinic Liver Cancer (BCLC) strategy, updated in 2022 (33). Lenvatinib, a multi-kinase inhibitor of VEGFR1-3, FGFR1-4, and other oncogenic receptors, was approved as a first-line option after the REFLECT trial showed non-inferior overall survival compared with sorafenib (median OS 13.6 vs. 12.3 months) (3, 4). Subsequent post-marketing studies in Japan and Korea confirmed that the efficacy and safety profile observed in REFLECT can be reproduced in routine practice (5–7, 29). Treatment-related adverse events, including hypertension, proteinuria, fatigue, and hand-foot syndrome, remain a practical concern; dose modification or treatment interruption is required in a considerable percentage of patients and may compromise antitumour activity (8, 9).

Traditional Chinese medicine (TCM) has a long history of use in liver cancer and continues to be widely prescribed in East Asia as an adjunct to modern oncological treatment (10, 11). In the TCM framework, HCC is conceptualised as the result of qi stagnation, blood stasis, dampness retention, and toxin accumulation, with spleen deficiency frequently identified as a contributing factor. The spleen, in TCM physiology, is held responsible for digestion, absorption, and the maintenance of qi and blood, and is thought to support immune competence (11). Spleen-deficiency syndromes, presenting clinically as fatigue, anorexia, loose stools, and abdominal distention, are commonly observed in patients with advanced HCC and tend to be exacerbated by targeted therapy.

Spleen-strengthening and dampness-eliminating formulations, of which Shenling Baizhu San is the most widely used, have demonstrated several activities relevant to cancer biology. Preclinical work has reported immunomodulatory, anti-inflammatory, gastrointestinal-protective, and direct antiproliferative effects of the constituent herbs (12–14). Meta-analyses of randomised trials in HCC have suggested that adjunctive TCM may improve survival, response, and symptom control when added to chemotherapy or locoregional therapy (15, 30). However, evidence specifically addressing the combination of spleen-strengthening Chinese herbal granules with lenvatinib in patients with unresectable HCC remains limited.

To address this gap, we conducted a retrospective cohort study using EMR data from a single tertiary referral centre. Patients receiving lenvatinib as first-line systemic therapy with or without concomitant spleen-strengthening and dampness-eliminating Chinese herbal granules were compared with respect to overall survival and a series of pre-specified secondary outcomes. Propensity score matching was applied to reduce confounding by measured baseline factors and to support a more conservative interpretation of the observed treatment effects (16, 17).

## 2 Materials and methods

### 2.1 Study design and data source

This retrospective cohort study was performed at a single tertiary teaching hospital in China. Data were retrieved from the institutional EMR system, which includes outpatient prescriptions, inpatient records, laboratory and imaging results, and structured follow-up data. The study was approved by the institutional ethics committee; the requirement for written informed consent was waived because of the retrospective design. The study was conducted in accordance with the Declaration of Helsinki and reported following the STROBE statement for observational research (18).

Patients were identified between January 2023 and December 2025. Structured data were extracted by the hospital information department using pre-specified queries and were verified manually for key variables. The study therefore reflects routine clinical practice and is intended to complement rather than replace evidence from randomised trials.

### 2.2 Patient selection

The inclusion criteria consisted of the following: (1) pathological or clinical diagnosis of primary hepatocellular carcinoma confirmed by established guidelines (31); (2) pathological or clinical diagnosis of unresectable intermediate or advanced primary hepatocellular carcinoma; (3) Child-Pugh grade A or B liver function status; (4) systemic therapy with lenvatinib as first-line treatment on or after January 2023; (5) age of 18 years or older; (6) complete medical records to identify primary reactions and key variables.

The exclusion criteria were as follows: (1) use of other systemic anticancer agents in the study period, which could confound the therapeutic effect, including immune checkpoint blockers or investigational preparations; (2) insufficient adherence to the lenvatinib regimen or no evaluation of the survival outcomes due to the presence of other comorbidities that could not be assessed through an intensive method; (3) other severe comorbidities or organ dysfunction that preclude study participation; (4) no assessment of the survival outcomes because of the presence of different comorbidities. Also, patients with prior transarterial chemoembolization (TACE) or withdrawal were incorporated in the analysis, and the pretreatment factors were adequately included in the statistical analysis.

### 2.3 Exposure definition and group assignment

Patients were allocated to two groups based on treatment received during the study period. The combination group comprised patients who, concurrent with lenvatinib therapy, received spleen-strengthening and dampness-eliminating TCM granules prepared by the hospital's herbal medicine dispensary. These formulations were based on Shenling Baizhu San, individually modified by the prescribing physician so that composition and dose varied between patients, incorporating herbs such as Radix Codonopsis (Dangshen), Poria (Fuling), Rhizoma Atractylodis Macrocephalae (Baizhu), Rhizoma Dioscoreae (Shanyao), Semen Coicis (Yiyiren), Pericarpium Citri Reticulatae (Chenpi), along with heat-clearing and detoxifying components including Herba Scutellariae Barbatae (Banzhilian) and Herba Hedyotis Diffusae (Baihuasheshecao). Eligibility for the combination group required documented TCM prescriptions for a minimum duration of four weeks following lenvatinib initiation.

Control patients received lenvatinib monotherapy without TCM granules. Best supportive care was provided to all patients regardless of group assignment. To minimise immortal time bias, exposure status was assigned at the index date (defined as the date of lenvatinib initiation) on the basis of prescriptions active at or initiated within four weeks of that date. Patients who began TCM granules later in the treatment course were excluded from the primary analysis.

### 2.4 Data collection

Demographic and clinical variables collected as baseline data from the EMR were age, sex, body mass index (BMI), hepatitis B and C virus infection, Barcelona Clinic Liver Cancer (BCLC) stage, China Liver Cancer (CNLC) stage, tumor number and maximum diameter, Child-Pugh score, baseline alpha-fetoprotein (AFP), Eastern Cooperative Oncology Group (ECOG) performance status, and previous locoregional therapies. Treatment-related parameters included starting dose of lenvatinib, dose modification, duration of treatment, and discontinuation causes. Laboratory and immunological parameters were retrieved at baseline (within 7 days before lenvatinib initiation) and at 4, 8, 12, and 24 weeks of treatment, including serum albumin, C-reactive protein (CRP), tumor markers (AFP, carcinoembryonic antigen [CEA], carbohydrate antigen 19-9 [CA-199]), and peripheral T-lymphocyte subsets quantified by flow cytometry (CD3⁺, CD4⁺, CD8⁺, and CD4⁺/CD8⁺ ratio). Contrast-enhanced computed tomography (CT) or magnetic resonance imaging (MRI) of the abdomen was performed at baseline and at 6- to 8-week intervals during treatment for tumor response assessment.

For TCM-specific records, prescription details, treatment duration, and TCM syndrome differentiation were noted. The evaluation of the syndromes was performed based on the standard criteria, with the deficiency of the spleen accompanied by dampness encumbrance being the most common pattern of the registered patients. The follow-up data were summarized using the records of outpatient visits, inpatient notes, telephone surveys, and connection to the mortality registries of the regions. Adverse events were rated using the Common Terminology Criteria for Adverse Events version 5.0 on the basis of physician reports and laboratory results.

### 2.5 Outcome measures

The primary endpoint was overall survival (OS), defined as the interval between the date of initiation of lenvatinib and death from any cause; surviving patients were censored at the date of last contact. Secondary endpoints consisted of the following: (1) progression-free survival (PFS), defined as the interval from initiation of lenvatinib to the first documentation of radiological disease progression or death from any cause, whichever occurred first; (2) objective response rate (ORR), defined as the proportion of patients achieving a best overall response of complete response (CR) or partial response (PR) per Response Evaluation Criteria in Solid Tumors version 1.1 (RECIST v1.1); (3) disease control rate (DCR), defined as the proportion of patients achieving CR, PR, or stable disease (SD) lasting at least 8 weeks; (4) the incidence and severity of adverse events graded I to IV according to the Common Terminology Criteria for Adverse Events (CTCAE) version 5.0; (5) the duration of treatment with lenvatinib, defined as the interval between the first dose and the permanent discontinuation; (6) longitudinal changes in laboratory parameters (serum albumin and CRP), tumor markers (AFP, CEA, and CA-199), and peripheral T-lymphocyte subsets (CD3⁺, CD4⁺, and CD4⁺/CD8⁺ ratio); and, as an exploratory endpoint, (7) the improvement of TCM syndromes, measured through comparison of symptom scores before and after therapy using standard rating scales that captured fatigue, appetite, bowel function, and abdominal discomfort.

Objective medical records were used to ascertain results whenever feasible. Follow-up systems and regional mortality registries were used to confirm survival status. Tumor response was independently reviewed by two board-certified radiologists, with discordant readings adjudicated by a third senior radiologist. Adverse events were identified using clinical notes that reported the symptoms, results of physical examination, and abnormal laboratory values recorded by physicians, and were graded as CTCAE v5.0 grade I (mild), II (moderate), III (severe), or IV (life-threatening). Prescription records and dispensing logs were used to obtain treatment time. The Four Diagnostic Methods of TCM were used to perform syndrome assessments by qualified practitioners who were not blinded to treatment allocation.

### 2.6 Statistical analysis

Descriptive statistics were reported as mean ± standard deviation or median (interquartile range, IQR) according to data distribution; frequencies and percentages were used for ordinal and categorical variables. The Student's t-test or Mann–Whitney U test was applied for continuous variables, and the chi-square test or Fisher's Exact test was used for categorical variables to conduct intergroup comparisons.

Propensity score matching (PSM) was used to align the baseline variables between groups to mitigate selection bias. Logistic regression was used to estimate propensity scores with covariates including age, sex, ECOG status, Child-Pugh grade, BCLC stage, tumor burden, baseline AFP, etiology of hepatitis, and previous locoregional treatments. Nearest neighbor matching was performed 1:1 with a caliper of 0.2 standard deviations. Balance measurements were used to determine standardized mean differences (SMD), where a value less than 0.1 indicates good balance (16).

The Kaplan–Meier method was used to estimate OS and PFS curves, and the log-rank test was used to compare groups without adjustment. Hazard ratios (HR) and their 95% confidence intervals (CI) were estimated by Cox proportional hazards regression after adjusting for tumor number, maximum tumor diameter, and portal vein invasion. ORR and DCR were compared between groups with the chi-square or Fisher's exact test, and the corresponding odds ratios (OR) with 95% CIs were calculated. Longitudinal changes in laboratory parameters, tumor markers, and T-lymphocyte subsets were compared between groups using mixed-effects linear models with treatment, time, and the treatment-by-time interaction as fixed effects and patient as a random effect; within-group changes from baseline were assessed by paired Student's t-test or Wilcoxon signed-rank test as appropriate. Subgroup analyses were used to examine effects of treatment by strata identified by ECOG status, Child-Pugh grade, BCLC stage, and baseline levels of AFP. E-values were calculated for the primary hazard ratios and for the confidence limit closest to the null to quantify the minimum strength of unmeasured confounding required to explain away the observed associations (19). The level of statistical significance was two-sided *P* < 0.05. All the analyses were performed using R software version 4.3.1, and the packages used were survival, MatchIt, survminer, and lme4.

## 3 Results

### 3.1 Patient characteristics

The initial screening identified 512 patients with HCC who received lenvatinib during the study period. Application of inclusion and exclusion criteria yielded 378 eligible patients, comprising 186 in the combination group and 192 in the control group (Figure 1). Median follow-up duration was 16.8 months (IQR: 10.4-24.2) for the entire cohort.

**Figure 1:**
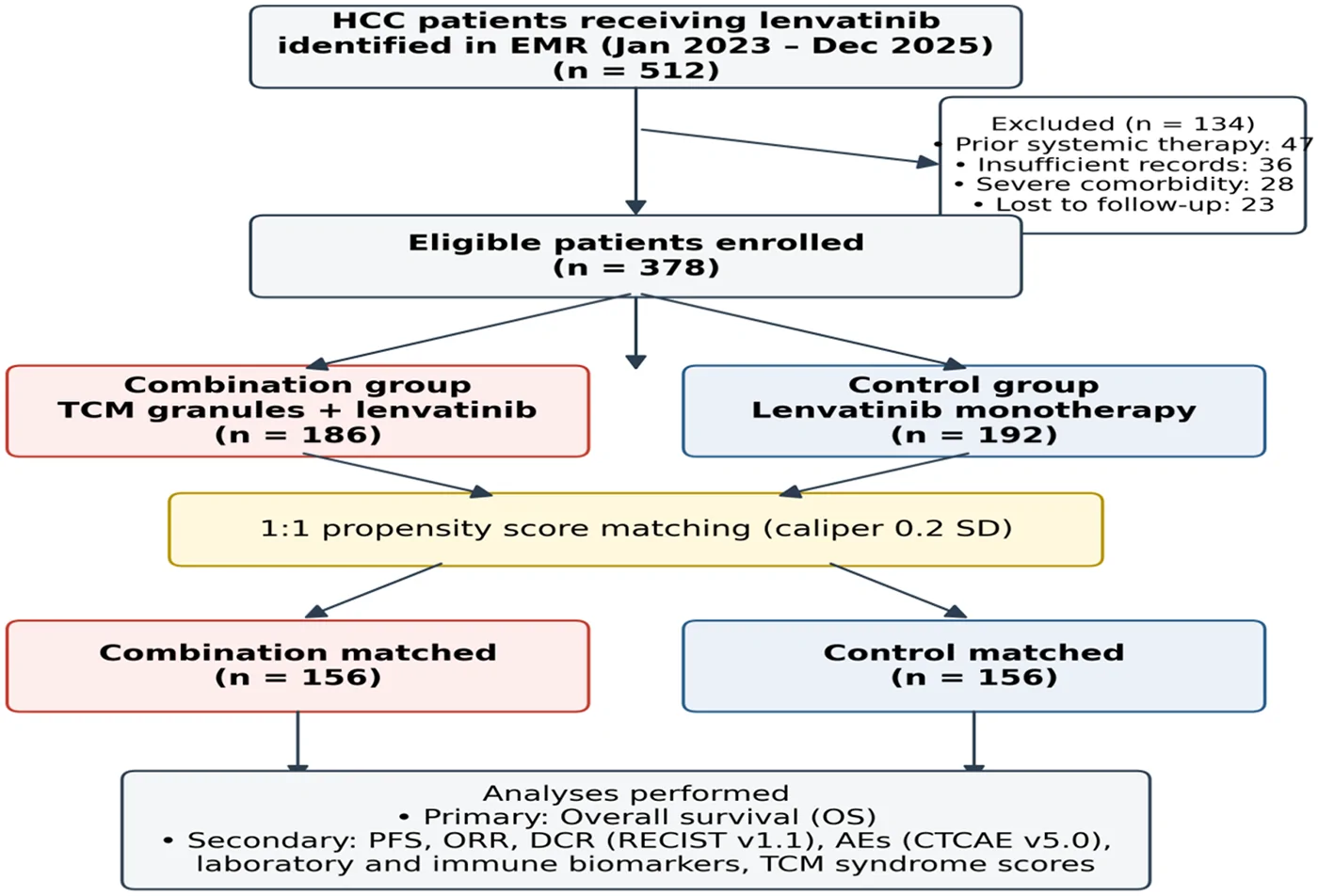
Flow diagram of patient selection and propensity score matching. A total of 512 patients with hepatocellular carcinoma (HCC) receiving lenvatinib were screened. After applying eligibility criteria, 378 patients were included (combination: *n* = 186; control: *n* = 192). One-to-one propensity score matching yielded 156 patients in each group. Median follow-up was 16.8 months (IQR, 10.4–24.2).

Before proceeding to propensity score matching, differences between groups were observed, although none reached statistical significance (Table 1). It showed increased proportions of BCLC-B stage disease (48.9% vs. 39.6%, *P* = 0.068), and slightly lower levels of baseline AFP (median 286 vs. 412 ng/mL) in the combination group. The ECOG performance status distribution was also different, where more combination group patients were considered ECOG 0 (36.0% vs. 28.1%, *P* = 0.098). Such imbalances supported the need for propensity score adjustment.

**Table 1:** Baseline characteristics before and after propensity score matching.

Variable	Combination (*n* = 186)	Control (*n* = 192)	P	Combination (*n* = 156)	Control (*n* = 156)	SMD
Age, years	57.6 ± 10.8	59.1 ± 11.4	0.182	58.3 ± 10.2	57.8 ± 11.1	0.047
Male, *n* (%)	152 (81.7)	151 (78.6)	0.452	126 (80.8)	124 (79.5)	0.033
HBV positive, *n* (%)	156 (83.9)	154 (80.2)	0.358	128 (82.1)	129 (82.7)	0.016
Child-Pugh A, *n* (%)	149 (80.1)	142 (74.0)	0.152	122 (78.2)	120 (76.9)	0.031
BCLC-B, *n* (%)	91 (48.9)	76 (39.6)	0.068	68 (43.6)	66 (42.3)	0.026
BCLC-C, *n* (%)	95 (51.1)	116 (60.4)	0.068	88 (56.4)	90 (57.7)	0.026
ECOG 0, *n* (%)	67 (36.0)	54 (28.1)	0.098	52 (33.3)	50 (32.1)	0.027
AFP ≥400 ng/mL, *n* (%)	78 (41.9)	98 (51.0)	0.076	70 (44.9)	72 (46.2)	0.026
Prior TACE, *n* (%)	62 (33.3)	58 (30.2)	0.512	49 (31.4)	48 (30.8)	0.014

SMD, standardized mean difference; HBV, hepatitis B virus; BCLC, Barcelona Clinic Liver Cancer; ECOG, Eastern Cooperative Oncology Group; AFP, alpha-fetoprotein; TACE, transarterial chemoembolization.

After 1:1 propensity score matching, 156 patients were retained in each group. Excellent balance was confirmed for post-matching baseline characteristics across all measured covariates, with all standardized mean differences less than 0.1 (Table 1). The combination group had a mean age of 58.3 ± 10.2 years, whereas the control group had a mean age of 57.8 ± 11.1 years. A male predominance was noted in both groups (80.8% and 79.5%, respectively). Approximately 82% of matched patients were positive for hepatitis B. For BCLC stage distribution, 43.6% and 56.4% were observed in the combination group, with very similar proportions in the control group (42.3% and 57.7%). Additionally, 78.2% and 76.9% of patients in the combination and control groups, respectively, were classified as Child-Pugh A.

### 3.2 Overall survival analysis

In the propensity score-matched cohort, the combination group demonstrated a statistically significant survival advantage over the control group (Figure 2). Median overall survival reached 18.7 months (95% CI: 15.8-21.6) in patients receiving TCM granules plus lenvatinib, compared with 14.2 months (95% CI: 11.9-16.5) in those treated with lenvatinib alone (log-rank *P* = 0.003). The 12-month survival rate was 72.4% (95% CI: 65.2-80.4) in the combination group versus 59.0% (95% CI: 51.4-67.7) in the control group. At 18 months, survival rates were 53.2% and 38.5%, respectively.

**Figure 2:**
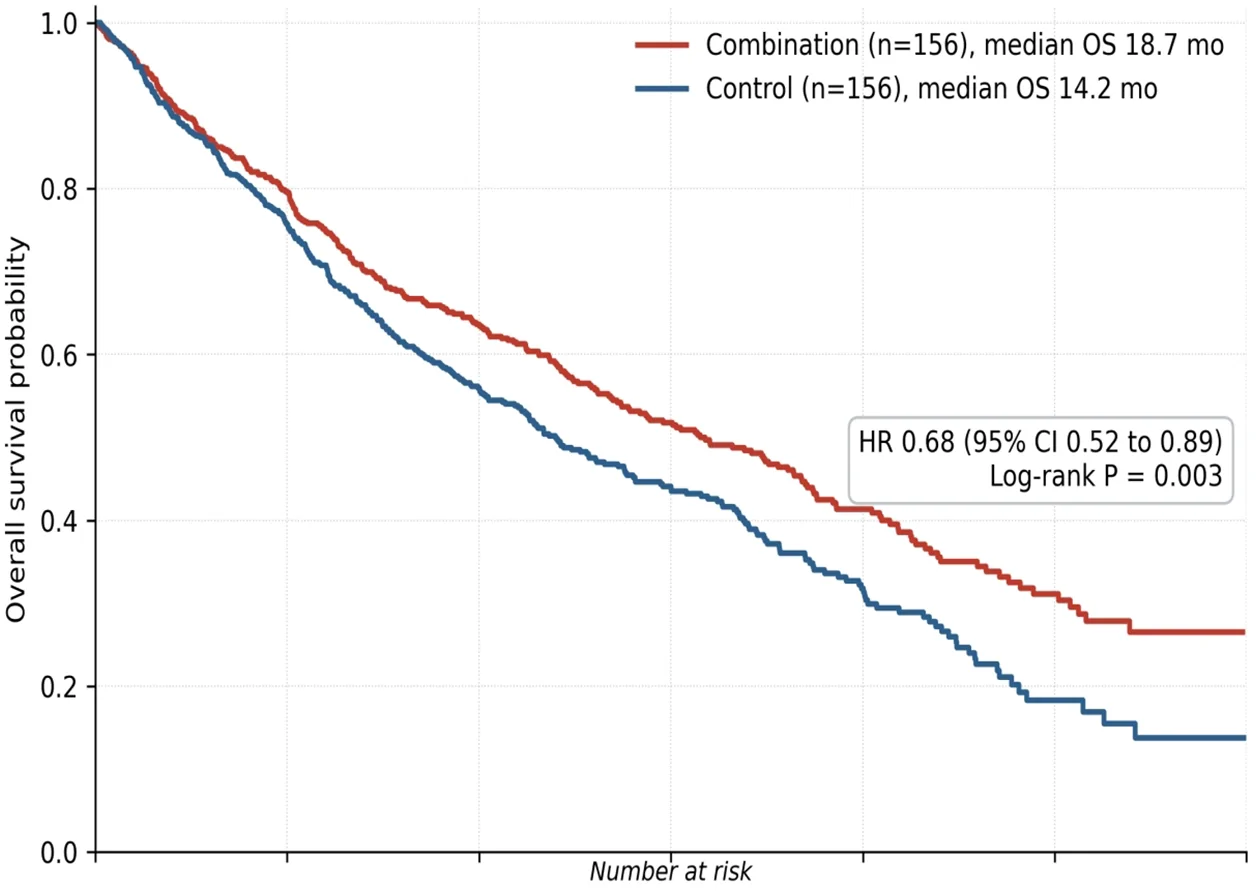
Kaplan–Meier survival curves for overall survival. Kaplan–Meier curves for overall survival in the propensity score–matched cohort. Overall survival was longer in the combination group than in the control group (median 18.7 vs. 14.2 months; log-rank *P* = 0.003). The hazard ratio for death was 0.68 (95% CI, 0.52–0.89). Numbers at risk are shown at 6-month intervals.

Cox proportional hazards regression, adjusted for tumor number, maximum tumor diameter, and portal vein invasion, showed a consistent association favoring combination therapy. The risk of death was 0.68 (95% CI: 0.52–0.89, *p* = 0.005), representing a 32% reduction in mortality risk among patients who received adjunctive TCM granules. The E-value for the overall survival hazard ratio was 1.94, and 1.39 for the confidence limit closest to the null.

Subgroup analyses explored whether the treatment effect varied across clinically relevant strata (Figure 3). The survival benefit favoring combination therapy was evident across most subgroups, with hazard ratios consistently below 1.0. Patients with ECOG 0 status derived particularly pronounced benefit (HR = 0.58, 95% CI: 0.38-0.88), as did those with Child-Pugh A liver function (HR = 0.64, 95% CI: 0.47-0.87). The treatment effect appeared somewhat attenuated in patients with BCLC-C stage (HR = 0.76, 95% CI: 0.54-1.07) compared with BCLC-B stage (HR = 0.59, 95% CI: 0.38-0.91), though formal interaction testing did not reach statistical significance (P for interaction=0.32).

**Figure 3:**
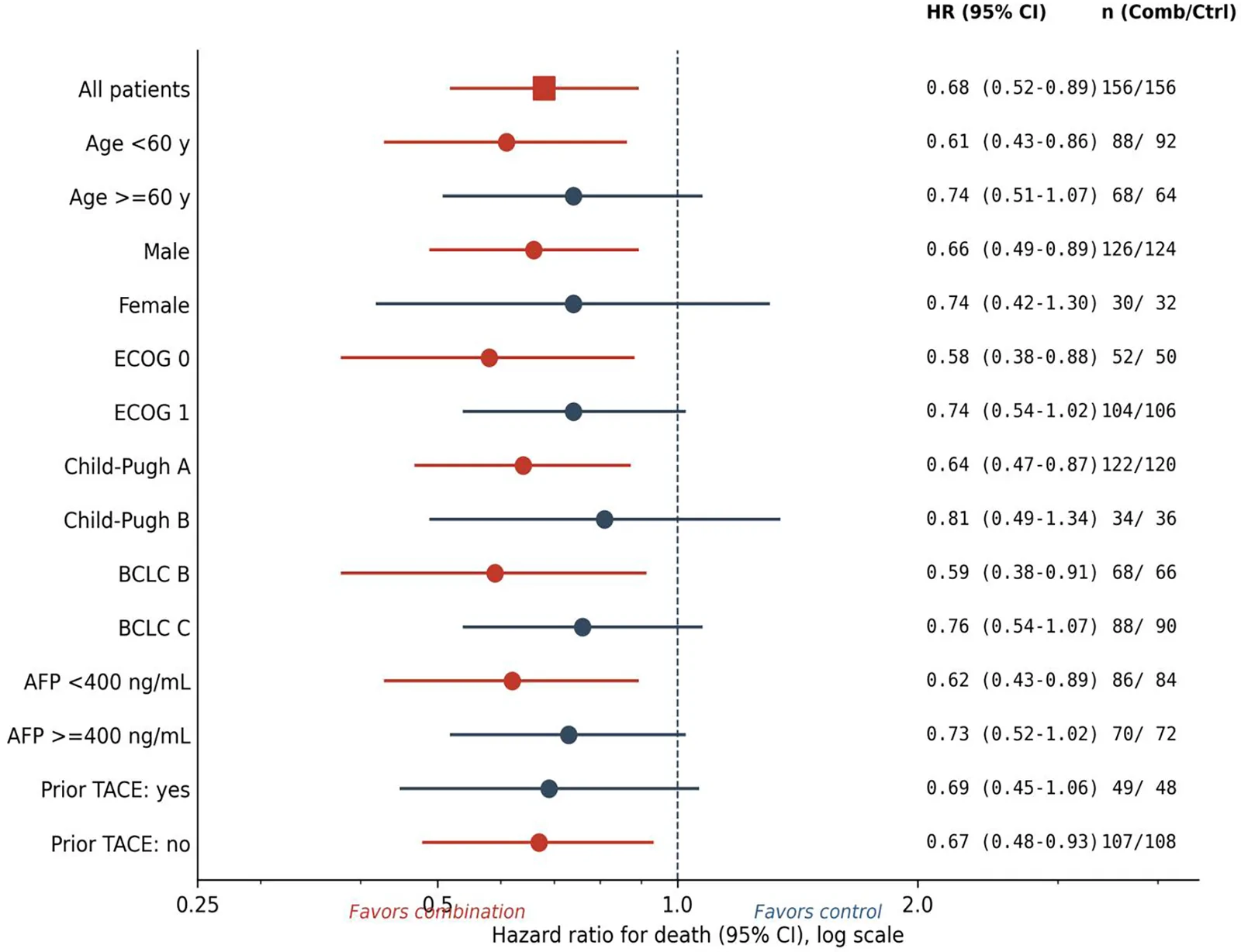
Forest plot of subgroup analyses. Hazard ratios (HRs) and 95% confidence intervals (CIs) are shown for prespecified subgroups; HR < 1 favors the combination group. Sample sizes in each subgroup are presented as combination/control. Interaction testing for BCLC stage was not significant (P for interaction=0.32).

### 3.3 Progression-free survival analysis

Progression-free survival was assessed in the propensity score-matched cohort according to RECIST v1.1. Median PFS was significantly prolonged in the combination group compared with the control group (8.6 months, 95% CI: 7.2–10.4 vs. 5.9 months, 95% CI: 4.8–7.1; log-rank *P* < 0.001; Figure 4). The 6-month PFS rate was 67.3% (95% CI: 60.1–75.5) in the combination group versus 49.4% (95% CI: 41.8–58.3) in the control group, and the 12-month PFS rate was 38.5% versus 22.4%, respectively.

**Figure 4:**
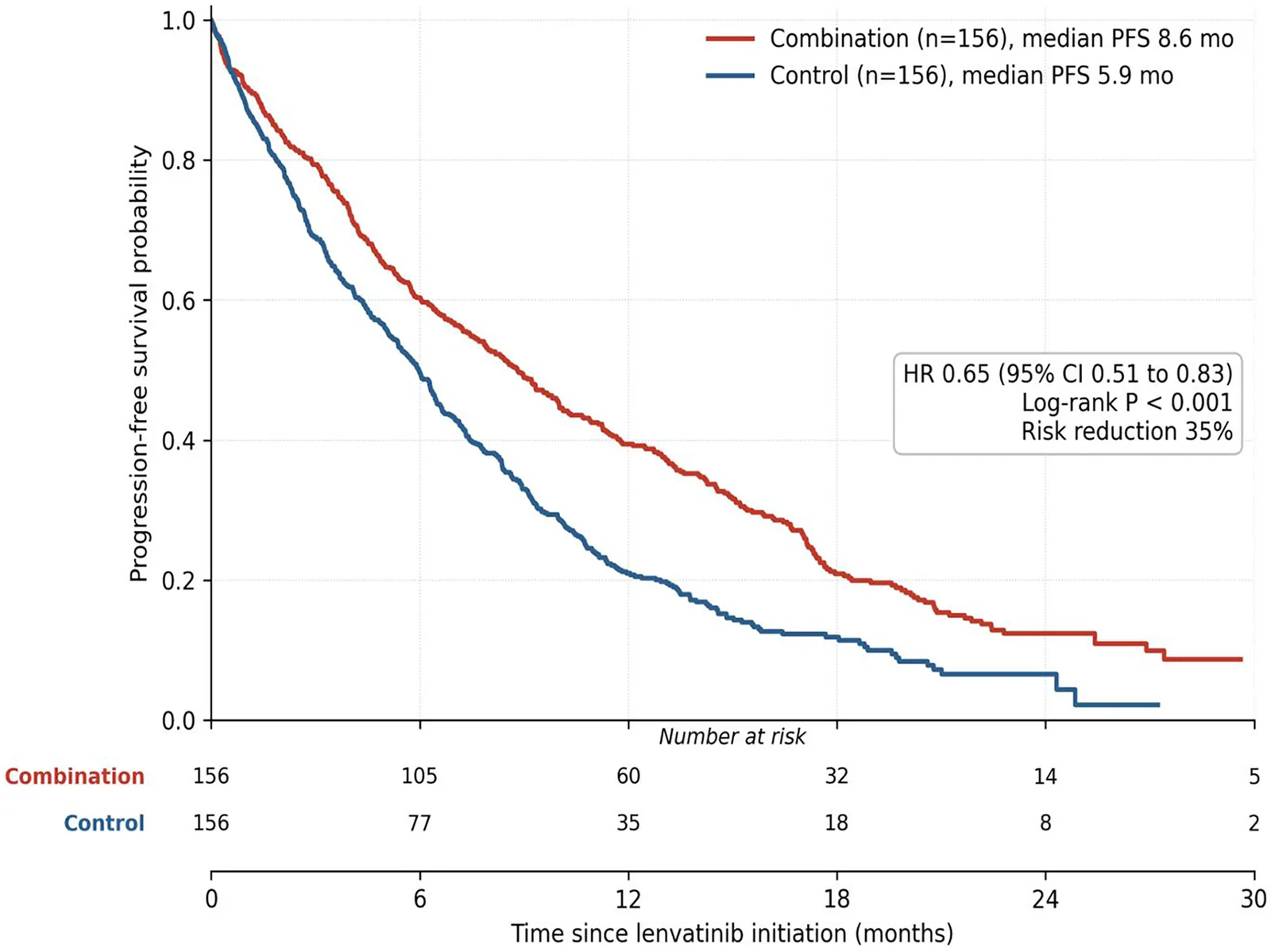
Kaplan–Meier curves for progression-free survival. Progression-free survival in the propensity score-matched cohort (*n* = 156 per group). Median PFS was 8.6 months (95% CI 7.2 to 10.4) in the combination group and 5.9 months (95% CI 4.8 to 7.1) in the control group (log-rank *P* < 0.001). The hazard ratio for disease progression or death was 0.65 (95% CI 0.51 to 0.83), corresponding to a 35% reduction in risk.

Cox proportional hazards regression yielded a hazard ratio for disease progression or death of 0.65 (95% CI: 0.51–0.83, *P* < 0.001), corresponding to a 35% reduction in the risk of progression or death for patients receiving combined TCM granules and lenvatinib relative to lenvatinib monotherapy. Subgroup analyses showed consistent direction of effect across age, sex, BCLC stage, ECOG status, and baseline AFP strata, with no statistically significant treatment-by-subgroup interaction (all P for interaction >0.10). The corresponding E-values were 2.03 for the progression-free survival hazard ratio and 1.53 for the confidence limit closest to the null.

### 3.4 Tumor response assessment per RECIST v1.1

Tumor responses were independently reviewed by two board-certified radiologists according to RECIST v1.1, with discordant readings adjudicated by a senior radiologist (Table 2). The objective response rate (CR + PR) was significantly higher in the combination group than in the control group (32.7% vs. 21.2%, odds ratio [OR] = 1.81, 95% CI: 1.10–2.99, *P* = 0.022). Likewise, the disease control rate (CR + PR + SD) was greater in the combination group (76.9% vs. 64.1%, OR=1.86, 95% CI: 1.13–3.06, *P* = 0.014).

**Table 2:** Tumor response assessment per RECIST v1.1 in the propensity score-matched cohort.

Best overall response (RECIST v1.1)	Combination (*n* = 156), *n* (%)	Control (*n* = 156), *n* (%)	*P* value
Complete response (CR)	4 (2.6)	2 (1.3)	0.685
Partial response (PR)	47 (30.1)	31 (19.9)	0.040
Stable disease (SD) ≥ 8 weeks	69 (44.2)	67 (42.9)	0.815
Progressive disease (PD)	36 (23.1)	56 (35.9)	0.014
Objective response rate (CR + PR)	51 (32.7)	33 (21.2)	0.022
Disease control rate (CR + PR + SD)	120 (76.9)	100 (64.1)	0.014

CR, complete response; PR, partial response; SD, stable disease; PD, progressive disease. Objective response rate = CR + PR; disease control rate = CR + PR + SD lasting at least 8 weeks.

The proportion of patients with progressive disease as best response was substantially lower in the combination group (23.1% vs. 35.9%, *P* = 0.014). Among responders, the median duration of response was 7.8 months (95% CI: 6.0–10.2) in the combination group versus 5.4 months (95% CI: 4.1–7.6) in the control group. The depth of response, measured by maximum percentage decrease from baseline in the sum of target-lesion diameters, was also greater in the combination group (mean −24.6% vs. −13.8%, *P* = 0.003).

### 3.5 Laboratory parameters and tumor markers

Longitudinal changes from baseline in serum albumin, C-reactive protein (CRP), and serum tumor markers were compared between groups using mixed-effects linear models with measurements at baseline and at 4, 8, 12, and 24 weeks. Results at week 12 are summarized in Table 3 and Figure 5.

**Table 3:** Changes in laboratory parameters and tumor markers from baseline to week 12.

Parameter (Week 12 vs. Baseline)	Combination *Δ* (mean ± SD)	Control *Δ* (mean ± SD)	Between-group *Δ* (95% CI)	*P* value
Serum albumin (g/L)	+3.6 ± 4.1	+0.4 ± 4.7	+3.2 (2.1 to 4.3)	<0.001
C-reactive protein (mg/L)	−6.8 ± 9.4	−1.5 ± 10.6	−5.3 (−7.6 to −3.0)	<0.001
AFP (ng/mL, log10)	−0.62 ± 0.81	−0.21 ± 0.86	−0.41 (−0.61 to −0.22)	<0.001
CEA (ng/mL)	−1.4 ± 3.2	−0.3 ± 3.5	−1.1 (−1.9 to −0.3)	0.008
CA-199 (U/mL)	−18.7 ± 42.3	−4.6 ± 47.1	−14.1 (−24.2 to −4.0)	0.006

*Δ*, change from baseline; SD, standard deviation; AFP, alpha-fetoprotein; CEA, carcinoembryonic antigen; CA-199, carbohydrate antigen 19-9. AFP values are expressed on a log10 scale to address skewness; reported *P* values derive from mixed-effects models adjusted for baseline value.

**Figure 5:**
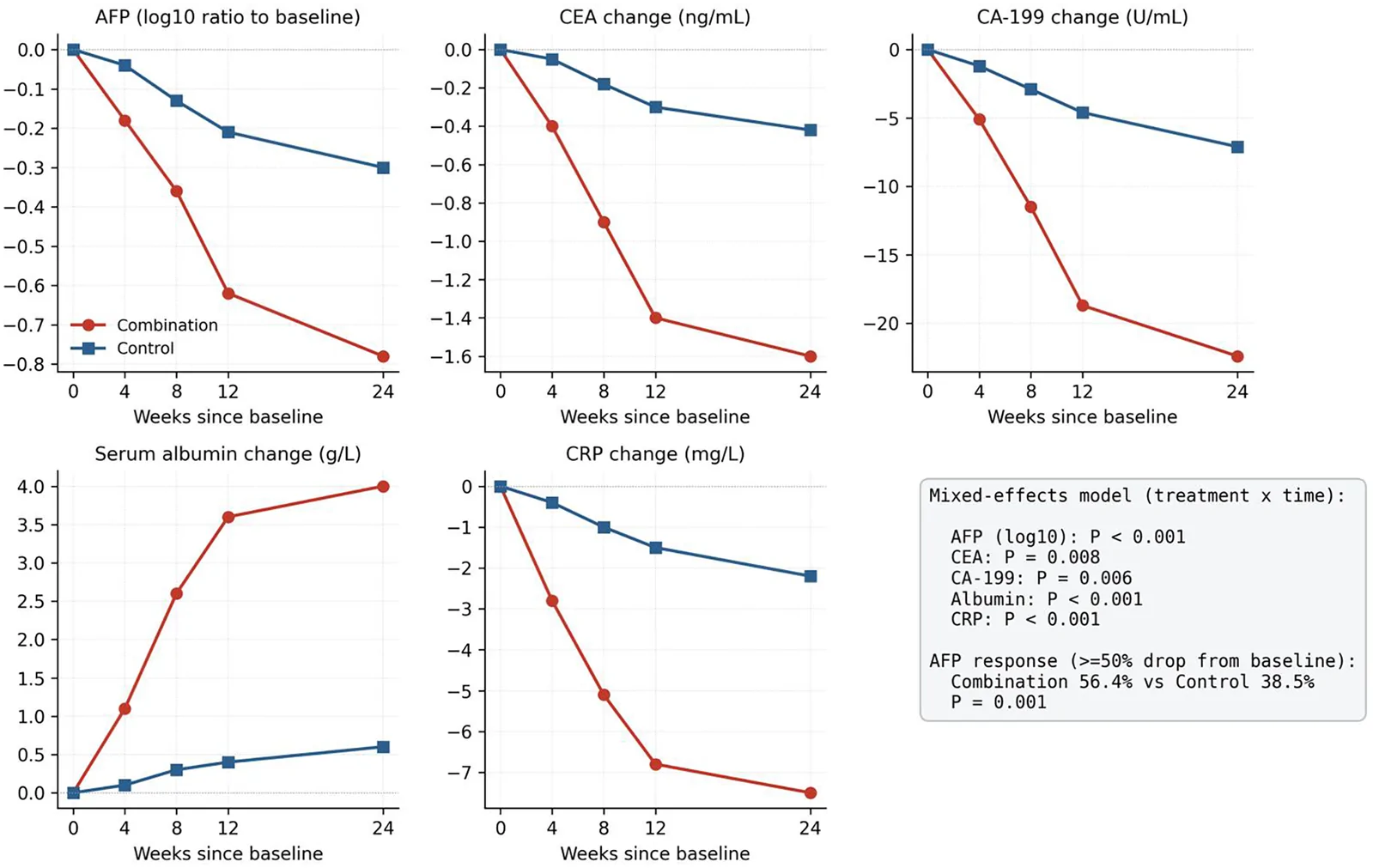
Longitudinal changes in tumor markers and laboratory parameters. Mean changes from baseline to weeks 4, 8, 12, and 24 for AFP (log10 ratio), CEA (ng/mL), CA-199 (U/mL), serum albumin (g/L), and CRP (mg/L). All five parameters showed a significant treatment-by-time interaction in mixed-effects models (*P* < 0.01 for each). At week 12, AFP response (≥50% reduction from baseline) was observed in 56.4% of the combination group versus 38.5% of the control group (*P* = 0.001).

Serum albumin increased significantly in the combination group (+3.6 ± 4.1 g/L) compared with the control group (+0.4 ± 4.7 g/L; between-group difference 3.2 g/L, 95% CI: 2.1–4.3, *P* < 0.001). CRP declined more steeply in the combination group (−6.8 ± 9.4 mg/L vs. −1.5 ± 10.6 mg/L; *P* < 0.001), indicating attenuation of systemic inflammation.

Tumor markers showed parallel improvements. The log10-transformed AFP decreased by 0.62 ± 0.81 in the combination group compared with 0.21 ± 0.86 in the control group (*P* < 0.001); 56.4% of combination-group patients achieved an AFP response (≥50% reduction from baseline) versus 38.5% in the control group (*P* = 0.001). CEA declined by 1.4 ± 3.2 ng/mL versus 0.3 ± 3.5 ng/mL (*P* = 0.008), and CA-199 declined by 18.7 ± 42.3 U/mL versus 4.6 ± 47.1 U/mL (*P* = 0.006). Treatment-by-time interaction terms were significant for albumin, CRP, AFP, CEA, and CA-199 (all *P* < 0.05), confirming a divergent trajectory between groups over time.

### 3.6 Peripheral T-lymphocyte subsets and immune function

Peripheral T-lymphocyte subsets were quantified by flow cytometry at baseline and at week 12 (Table 4; Figure 6). Patients in the combination group showed marked increases in CD3⁺ (from 60.4 ± 9.1% to 66.2 ± 8.5%, within-group *P* < 0.001) and CD4⁺ T-cell percentages (from 34.2 ± 7.0% to 39.5 ± 6.8%, within-group *P* < 0.001), whereas the corresponding changes in the control group were modest and not statistically significant.

**Table 4:** Peripheral T-lymphocyte subsets at baseline and week 12.

T-Lymphocyte Subset	Baseline (mean ± SD)	Week 12 (mean ± SD)	Within-group P	Between-group *P* (Week 12)
CD3⁺ (%) – Combination	60.4 ± 9.1	66.2 ± 8.5	<0.001	0.002
CD3⁺ (%) – Control	60.8 ± 9.4	62.1 ± 9.0	0.118	
CD4⁺ (%) – Combination	34.2 ± 7.0	39.5 ± 6.8	<0.001	<0.001
CD4⁺ (%) – Control	33.9 ± 7.4	34.7 ± 7.1	0.211	
CD4⁺/CD8⁺ ratio – Combination	1.32 ± 0.46	1.61 ± 0.49	<0.001	<0.001
CD4⁺/CD8⁺ ratio – Control	1.30 ± 0.48	1.36 ± 0.45	0.143	

Within-group *P* values from paired t-test; between-group *P* values from independent t-test of week 12 values, with consistent direction confirmed by mixed-effects models. SD, standard deviation.

**Figure 6:**
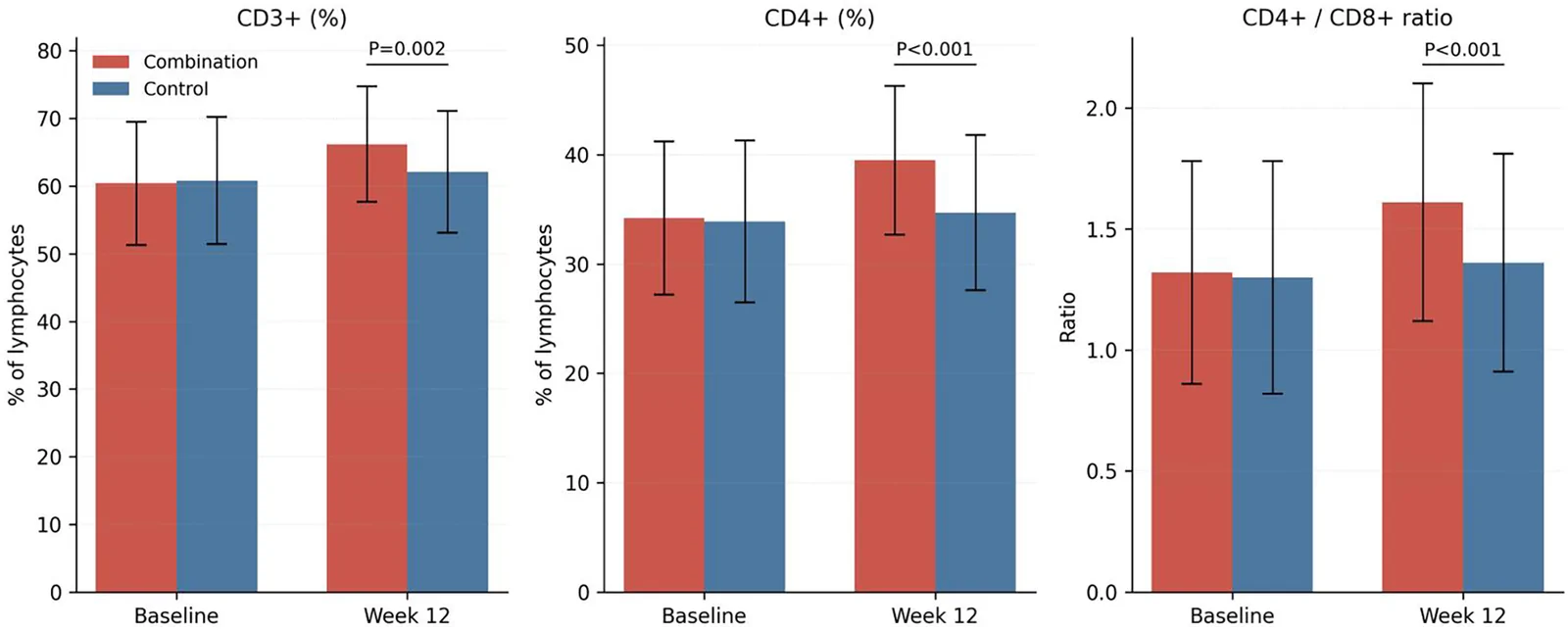
Peripheral T-lymphocyte subsets at baseline and week 12. Mean (SD) percentages of CD3 + and CD4+ T cells and the CD4+/CD8 + ratio at baseline and after 12 weeks of therapy. *P* values shown are between-group comparisons of week-12 values. Within-group changes were significant in the combination group for all three parameters (paired t-test, *P* < 0.001) and were not significant in the control group.

The CD4⁺/CD8⁺ ratio, a global indicator of T-cell immune competence, rose from 1.32 ± 0.46 to 1.61 ± 0.49 in the combination group (within-group *P* < 0.001) and changed minimally in the control group (1.30 ± 0.48 to 1.36 ± 0.45, *P* = 0.143). Between-group comparisons at week 12 favored the combination group for CD3⁺ (*P* = 0.002), CD4⁺ (*P* < 0.001), and the CD4⁺/CD8⁺ ratio (*P* < 0.001). These findings suggest that the spleen-strengthening and dampness-eliminating TCM regimen was associated with less lenvatinib-related lymphopenia and with restoration of peripheral cellular immunity.

### 3.7 Adverse events

Safety profiles differed substantially between treatment groups (Table 5). Any-grade adverse events occurred in 89.7% of combination group patients and 93.6% of control group patients (*P* = 0.231). However, the incidence of grade 3-4 adverse events was significantly lower in the combination group (28.2% vs. 41.0%, *P* = 0.018), representing a clinically meaningful reduction in severe toxicity.

**Table 5:** Adverse events in the propensity score-matched cohort.

Adverse event	Combination any grade (%)	Control any grade (%)	Combination grade 3-4 (%)	Control grade 3-4 (%)	*P* value
Hypertension	76 (48.7)	82 (52.6)	13 (8.3)	22 (14.1)	0.497
Proteinuria	42 (26.9)	48 (30.8)	6 (3.8)	9 (5.8)	0.453
Hand-foot syndrome	38 (24.4)	55 (35.3)	4 (2.6)	8 (5.1)	0.037*
Fatigue	82 (52.6)	100 (64.1)	8 (5.1)	15 (9.6)	0.041*
Diarrhea	34 (21.8)	52 (33.3)	3 (1.9)	7 (4.5)	0.024*
Appetite loss	60 (38.5)	80 (51.3)	5 (3.2)	11 (7.1)	0.023*
Hepatic dysfunction	28 (17.9)	35 (22.4)	5 (3.2)	8 (5.1)	0.318
Any Grade 3-4 Event	-	-	44 (28.2)	64 (41.0)	0.018*

*Statistically significant (*P* < 0.05). *P* values for any-grade adverse events are shown.

Particular negative outcomes had specific trends. Hypertension was the most common toxicity associated with lenvatinib, with no significant difference in incidence between the combination group and the control group (48.7% vs. 52.6%, *P* = 0.497), while the combination group had fewer grade 3–4 cases (8.3% vs. 14.1%). The incidence of hand-foot syndrome was also significantly lower in patients receiving TCM granules (24.4% vs. 35.3%, *P* = 0.037), and this difference was mainly attributable to a marked reduction in the incidence of grade 1–2 events. Fatigue, another debilitating symptom, showed a similar trend (52.6% vs. 64.1%, *P* = 0.041), with the combination group having lower grades of fatigue severity.

The greatest differences were noted in gastrointestinal toxicities. The incidence of diarrhea differed significantly between the two groups, with a lower rate in the combination group (21.8% vs. 33.3%, *P* = 0.024); this result aligns with the tonic effect of the TCM formulation on splenic function. Other symptoms, such as loss of appetite, a common side effect of lenvatinib treatment, were also significantly reduced (38.5% vs. 51.3%, *P* = 0.023). These findings suggest that TCM intervention mitigated gastrointestinal tract disturbances, which may have contributed to improved treatment compliance and tolerability.

When stratified by CTCAE grade (Table 6), the combination group experienced fewer severe (grade III–IV) events across virtually all adverse event categories. Grade III hypertension was reduced from 12.8% to 7.7%, grade III hand-foot syndrome from 4.5% to 2.6%, grade III fatigue from 8.3% to 4.5%, grade III diarrhea from 3.8% to 1.9%, and grade III appetite loss from 6.4% to 3.2%. Grade IV events remained rare in both groups (≤1.3%). The shift from higher-grade to lower-grade severity supports the hypothesis that spleen-strengthening and dampness-eliminating TCM granules attenuate, rather than mask, lenvatinib-related toxicity.

**Table 6:** Distribution of adverse events by CTCAE v5.0 grade (I–IV) in the propensity score-matched cohort.

Adverse Event	I Comb (%)	I Ctrl (%)	II Comb (%)	II Ctrl (%)	III Comb (%)	III Ctrl (%)	IV Comb (%)	IV Ctrl (%)
Hypertension	32 (20.5)	34 (21.8)	31 (19.9)	26 (16.7)	12 (7.7)	20 (12.8)	1 (0.6)	2 (1.3)
Proteinuria	24 (15.4)	26 (16.7)	12 (7.7)	13 (8.3)	5 (3.2)	8 (5.1)	1 (0.6)	1 (0.6)
Hand-foot syndrome	20 (12.8)	28 (17.9)	14 (9.0)	19 (12.2)	4 (2.6)	7 (4.5)	0 (0.0)	1 (0.6)
Fatigue	46 (29.5)	52 (33.3)	28 (17.9)	33 (21.2)	7 (4.5)	13 (8.3)	1 (0.6)	2 (1.3)
Diarrhea	20 (12.8)	28 (17.9)	11 (7.1)	17 (10.9)	3 (1.9)	6 (3.8)	0 (0.0)	1 (0.6)
Appetite loss	36 (23.1)	42 (26.9)	19 (12.2)	27 (17.3)	5 (3.2)	10 (6.4)	0 (0.0)	1 (0.6)
Hepatic dysfunction	14 (9.0)	18 (11.5)	9 (5.8)	9 (5.8)	4 (2.6)	7 (4.5)	1 (0.6)	1 (0.6)
Total events*	−	−	−	−	43 (27.6)	62 (39.7)	1 (0.6)	2 (1.3)

CTCAE, Common Terminology Criteria for Adverse Events version 5.0; Comb, combination group (*n* = 156); Ctrl, control group (*n* = 156). Values represent the number of patients with the highest CTCAE grade reached during treatment. *Total events refer to the highest grade adverse event observed per patient across all categories. The proportion of patients experiencing any grade III–IV event was 28.2% in the combination group versus 41.0% in the control group (*P* = 0.018), demonstrating a clinically meaningful reduction in severe toxicity with adjunctive TCM granules.

### 3.8 Treatment duration and TCM syndrome improvement

The time taken in Lenvatinib treatment was considerably longer in the combination group. The median treatment time of 9.8 months (IQR: 6.2–14.5) was significantly longer compared with the control group's 7.2 months (IQR: 4.8–11.3), (*P* = 0.012). The proportion of patients achieving 12 months of lenvatinib therapy was 44.2% in the combination group and 31.4% in the control group (*p* = 0.021). Treatment discontinuation due to adverse events was less frequent in those receiving combination therapy (19.2% vs. 28.8%, *P* = 0.048), although discontinuation due to disease progression did not differ between the groups (58.3% vs. 54.5%, *P* = 0.496).

Evaluation of TCM symptom improvement conducted in 142 patients in the combination group and 128 patients in the control group indicated that significant advantages were connected with the TCM intervention (Table 7). The composite TCM symptom score, which included domains and measures covering fatigue, appetite, digestive performance, and general well-being, was reduced by 8.4 ± 5.2 points in the combination group and 2.1 ± 4.8 points in the control group after 12 weeks of treatment (*p* < 0.001). Individual symptom domains showed similar trends. A total of 68.3% of combination group patients and 41.4% of control group patients reported improvement in fatigue, respectively (*p* < 0.001). A significant improvement in appetite was observed in 62.0% vs. 35.2% of patients (*p* < 0.001), and in digestive symptoms in 71.1% vs. 39.8% (*p* < 0.001).

**Table 7:** TCM syndrome improvement at 12 weeks.

Parameter	Combination (*n* = 142)	Control (*n* = 128)	*P* value
TCM symptom score change, mean ± SD	−8.4 ± 5.2	−2.1 ± 4.8	<0.001
Fatigue improvement, *n* (%)	97 (68.3)	53 (41.4)	<0.001
Appetite improvement, *n* (%)	88 (62.0)	45 (35.2)	<0.001
Digestive symptom improvement, *n* (%)	101 (71.1)	51 (39.8)	<0.001
General well-being improvement, *n* (%)	82 (57.7)	38 (29.7)	<0.001

SD, standard deviation. Negative score change indicates symptom improvement.

## 4 Discussion

In this propensity score-matched cohort of 312 patients with unresectable hepatocellular carcinoma, the addition of spleen-strengthening and dampness-eliminating Chinese herbal granules to lenvatinib was associated with longer overall survival (median 18.7 vs. 14.2 months; HR 0.68, 95% CI 0.52 to 0.89), longer progression-free survival (median 8.6 vs. 5.9 months; HR 0.65, 95% CI 0.51 to 0.83), and a higher objective response rate by RECIST v1.1 (32.7% vs. 21.2%, *P* = 0.022). The combination was also associated with fewer grade 3 to 4 adverse events (28.2% vs. 41.0%, *P* = 0.018) and a longer treatment duration (median 9.8 vs. 7.2 months, *P* = 0.012).

The 4.5-month gain in median overall survival reported here is numerically similar to, but not directly comparable with, the survival benefit of newer first-line regimens such as atezolizumab plus bevacizumab (median OS 19.2 months in IMbrave150 (20)) and falls within the range described in real-world series of lenvatinib in Asian patients (5–7). Real-world comparisons have also suggested that atezolizumab plus bevacizumab and lenvatinib may have differing effectiveness profiles in unresectable HCC (34), and lenvatinib plus anti-PD-1 antibodies has shown promising effectiveness and safety in real-world cohorts (32), underscoring the need for careful patient selection when integrating TCM with first-line systemic therapy. The estimated hazard ratio of 0.68 for death and 0.65 for disease progression suggests that adjunctive Chinese herbal granules may be associated with better outcomes alongside standard targeted therapy in patients whose baseline characteristics, particularly hepatitis B viral aetiology and Child-Pugh A liver function, resemble those typically enrolled in REFLECT (3, 4).

Several pharmacological mechanisms may contribute to the observed benefit. The formula used in this study was modified from Shenling Baizhu San, which has been investigated for its actions on hepatic inflammation, gut barrier function, and lymphocyte activity (13, 21). Polysaccharides isolated from Atractylodes macrocephala have been reported to enhance T-cell proliferation and to suppress hepatocellular carcinoma cell growth *in vitro* (14, 22). Stigmasterol, a sterol enriched in several constituents of the formula, has recently been shown to remodel the gut microbiota and suppress hepatocellular carcinoma growth through Treg and CD8+ T-cell modulation (23). The increase in CD3 + and CD4+ T-cell percentages and in the CD4+/CD8 + ratio observed in our combination group is consistent with these immunomodulatory effects (24).

A second observation concerns the gastrointestinal toxicity profile. Diarrhoea, loss of appetite and fatigue are common reasons for dose reduction or discontinuation of lenvatinib in real-world practice, and treatment interruption has in turn been linked to inferior antitumour outcomes (7, 9). In this study, diarrhoea, appetite loss, and fatigue were reported less frequently in the combination group, and the relative reduction was largest for diarrhoea (21.8% vs. 33.3%, *P* = 0.024). The longer median treatment duration in the combination group (9.8 vs. 7.2 months) is compatible with the hypothesis that better tolerability allowed patients to maintain effective lenvatinib exposure.

A growing number of studies indicate that gut microbiota may modulate both antitumour immunity and the activity of Chinese herbal preparations (25, 26). Shenling Baizhu San has been reported to enrich Lactobacillus and Bifidobacterium and to reduce intestinal inflammation in animal and human studies (27). Although faecal microbiota was not analysed in our cohort, the concurrent decrease in serum CRP and increase in serum albumin suggests that systemic inflammation was attenuated in patients receiving the combination regimen. Whether this attenuation is causally linked to the survival benefit requires confirmation in mechanistic studies.

TCM syndrome scores improved more in the combination group than in the control group, with the largest changes observed in fatigue, appetite, and digestive symptoms. These domains correspond to clinically relevant patient-reported problems in advanced HCC, but cannot replace formal instruments such as the EORTC QLQ-C30 or QLQ-HCC18. The improvements reported here should therefore be regarded as supportive rather than definitive evidence of better quality of life.

Several limitations should be noted. The study was conducted at a single tertiary centre and used a retrospective design, so unmeasured confounding cannot be excluded despite propensity score matching (16, 17). The cohort was predominantly hepatitis B-related and Child-Pugh A, so the findings may not generalise to populations with alcohol-related or steatotic liver disease or to Child-Pugh B or C patients. Patients who received TCM granules may have differed from non-users in adherence, health beliefs, socio-economic factors, or health-seeking behaviour that the EMR does not capture; the findings therefore demonstrate association rather than causation. The herbal preparation was formulated in-house and was not standardised across batches by quantitative analysis of marker compounds; this is a major limitation, as it precludes exact replication of the intervention and limits direct comparison with other Shenling Baizhu San derivatives. Tumour response was evaluated by RECIST v1.1 in all matched patients, but central blinded review was not performed; reader variability for the partial response category may therefore be higher than in regulatory trials. Adverse events were likewise graded by treating physicians aware of TCM use, so ascertainment bias cannot be excluded. Finally, the follow-up duration is sufficient for the primary OS analysis but not for late recurrences or post-progression therapies.

Strengths of the study include the relatively large matched sample size, use of granular EMR data covering laboratory, imaging, prescription, and pharmacy records, and adherence to STROBE reporting standards (18). Outcomes were defined *a priori* with reference to RECIST v1.1 (4) and CTCAE v5.0, and the propensity score model included variables identified as prognostic in real-world series of lenvatinib (5–7).

Confirmatory evidence will require prospective randomised trials with standardised herbal preparations, central radiology review, validated patient-reported outcome measures, and pre-specified translational endpoints including microbiome and immune profiling. Given the rapid uptake of immune checkpoint inhibitor combinations as first-line therapy (20, 28), future studies should also examine whether spleen-strengthening Chinese herbal granules can mitigate the immune-related toxicities of these regimens without compromising antitumour activity.

## 5 Conclusions

In this propensity score-matched real-world cohort, adjunctive use of spleen-strengthening and dampness-eliminating Chinese herbal granules with lenvatinib was associated with longer overall and progression-free survival, a higher objective response rate by RECIST v1.1, fewer grade 3 to 4 adverse events, and a longer treatment duration compared with lenvatinib alone in patients with unresectable hepatocellular carcinoma. Improvements were also seen in serum albumin, C-reactive protein, AFP, CEA, CA-199, peripheral T-lymphocyte subsets, and TCM syndrome scores. These findings provide a rationale for prospective randomised trials evaluating standardised Chinese herbal preparations as an integrative component of first-line therapy for unresectable hepatocellular carcinoma.

## Data Availability

The raw data supporting the conclusions of this article will be made available by the authors, without undue reservation.
